# 532. Protocols for High-voltage Upper Extremity Electrical Burns at level 1 Trauma Centers: A Scoping Review

**DOI:** 10.1093/jbcr/irag033.229

**Published:** 2026-04-06

**Authors:** Cole Stephany, Natalie K Blanc, Jenna Adalbert, Gil Gontre, Seth D Dodds

**Affiliations:** Miami Burn Center, Miami, FL; University of Miami Miller School of Medicine, Miami, FL; Miami Burn Center, Miami, FL; University of Miami, Miami, FL; University of Miami, Department of Orthopaedics, Miami, FL

## Abstract

**Introduction:**

High-voltage electrical burns of the upper extremity are rare yet devastating injuries that often require complex reconstructive interventions or limb amputation. Despite improved burn care over recent decades, no standardized surgical protocol exists, and the optimal timing and type of intervention remains controversial. This scoping review aims to synthesize current evidence on surgical and rehabilitative strategies in order to explore best practices and existing protocols for upper extremity burn management at Level 1 trauma centers.

**Methods:**

A scoping review of the literature was performed in PubMed, Ovid MEDLINE, and Scopus databases and restricted to English-language studies published from 2000 onward. Articles were included if they provided the following: (1) high-voltage (>1000 V) upper extremity electrical burns, (2) detailed surgical interventions preceding amputation, and (3) outcomes including amputation rates or flap success. Exclusion criteria included case reports without intervention details or studies on low-voltage injuries. Data were extracted on injury characteristics, surgical protocols, timing to intervention, and clinical outcomes. The Joanna Briggs Institute's critical appraisal tools were then used as a final measure to perform quality assessments of included articles.

**Results:**

Searches generated 58 publications, six eligible for analysis. For early decompression, a midline fasciotomy within 8 hours had a 27.8% amputation rate versus 57.0% with the conventional volar-ulnar technique. Both methods showed lower rates with early versus late (>8 hr) intervention. Among studies reporting flap failure, 89% occurred 5–21 days post-burn, while none occurred with delayed reconstructions after 6 weeks. Flap choice varied by injury extent, but in high-voltage burns, muscular flaps were most viable for amputation prevention.

**Conclusions:**

There is a paucity of literature that exists on standardized protocols for high-voltage upper extremity burn injuries. Current studies suggest that a decompressive midline fasciotomy within 8 hours of injury followed by delayed flap closure at a minimum of 3 weeks may reduce the requirement for amputation in this population. However, further studies are required to create an evidence-based protocol with specific time to intervention and management recommendations for high-voltage upper extremity burns.

**Applicability of Research to Practice:**

High-voltage electrical burns often cause progressive tissue damage that may not be apparent on initial examination. Clinically, early recognition and timely intervention are essential to reduce amputation rates and long-term morbidity. By analyzing prior management strategies, clinicians can refine protocols to guide surgical and reconstructive choices, ultimately improving limb salvage and functional recovery in these complex injuries.

**Funding for the study:**

N/A.

